# GABA_A_ receptor-acting neurosteroids: A role in the development and regulation of the stress response

**DOI:** 10.1016/j.yfrne.2014.06.001

**Published:** 2015-01

**Authors:** Benjamin G. Gunn, Linda Cunningham, Scott G. Mitchell, Jerome D. Swinny, Jeremy J. Lambert, Delia Belelli

**Affiliations:** a Division of Neuroscience, Medical Research Institute, Dundee University, Ninewells Hospital and Medical School, Dundee DD1 9SY, UK; b Institute for Biomedical and Biomolecular Sciences, School of Pharmacy and Biomedical Sciences, University of Portsmouth, UK

**Keywords:** Allopregnanolone, GABA_A_ receptor, HPA axis, Early-life stress, Depression

## Abstract

Regulation of hypothalamic–pituitary–adrenocortical (HPA) axis activity by stress is a fundamental survival mechanism and HPA-dysfunction is implicated in psychiatric disorders. Adverse early life experiences, e.g. poor maternal care, negatively influence brain development and programs an abnormal stress response by encoding long-lasting molecular changes, which may extend to the next generation. How HPA-dysfunction leads to the development of affective disorders is complex, but may involve GABA_A_ receptors (GABA_A_Rs), as they curtail stress-induced HPA axis activation. Of particular interest are endogenous neurosteroids that potently modulate the function of GABA_A_Rs and exhibit stress-protective properties. Importantly, neurosteroid levels rise rapidly during acute stress, are perturbed in chronic stress and are implicated in the behavioural changes associated with early-life adversity. We will appraise how GABA_A_R-active neurosteroids may impact on HPA axis development and the orchestration of the stress-evoked response. The significance of these actions will be discussed in the context of stress-associated mood disorders.

## Highlights

• GABA_A_ receptors (GABA_A_Rs) curtail stress-induced activation of the HPA axis.
• Stressful challenges evoke *de novo* brain synthesis of GABA_A_R-active neurosteroids (NS).
• NS inhibit the output of CRF-releasing neurones of the hypothalamus.
• NS actions in the hypothalamus are blunted in rodent models of early-life adversity.
• NS may be important molecular messengers in the programming of the stress-response.

## 1 Introduction

### 1.1 Stress, GABA_A_ receptors and neurosteroids

Stressful experiences engage a co-ordinated neuronal and hormonal response, orchestrated by the hypothalamic–pituitary–adrenocortical (HPA) axis *via* activation of corticotrophin releasing factor (CRF)-releasing parvocellular neurones of the hypothalamic paraventricular nucleus (PVN). The activity of the PVN is subject to regulation by GABA, the dominant inhibitory neurotransmitter in the hypothalamus (Decavel and Van den Pol, 1990; Miklos and Kovacs, 2002), which acts primarily *via* GABA_A_ receptors (GABA_A_Rs). The neurocircuitry regulating the activity of the PVN is highly complex, comprised of mono- and polysynaptic inputs from a number of different limbic and forebrain regions. GABA_A_Rs are expressed throughout this circuit where they play an important role in modulating the functional activity, and hence output, of these brain regions. Thus, regulation of HPA axis activity through GABA_A_R-mediated transmission not only occurs at the level of the PVN, but also at multiple levels of the stress neurocircuitry.

GABA_A_Rs possess a pentameric structure formed from multiple subunits. To date, 19 subunits have been identified (α1-6, β1-3, γ1-3, δ, ε, θ, π and ρ1-3), which are divided into subfamilies based upon their amino acid homology (Olsen and Sieghart, 2008, 2009). These subunits exhibit discrete expression profiles, allowing for the expression of ∼20–30 different GABA_A_R isoforms within the CNS (Fritschy and Brunig, 2003; Olsen and Sieghart, 2008; Hortnagl et al., 2013; Fritschy and Panzanelli, 2014) with most native receptors comprising two α, two β and a single γ, δ or ε subunit. Importantly, GABA_A_R isoforms containing the γ subunit are generally, albeit not exclusively (e.g. α5βγ2 isoforms) targeted to synapses where they mediate “phasic” GABAergic transmission, while δ-GABA_A_Rs comprise a major class of peri- and extrasynaptic receptors that mediate a “tonic” (Farrant and Nusser, 2005; Belelli et al., 2009) and “spill-over” (Herd et al., 2013) form of GABAergic inhibition. The subunit composition not only determines the regional and cellular location of GABA_A_Rs, but also influences their biophysical and pharmacological profile. For example, incorporation of the γ2 subunit in conjunction with specific α subunits (α1-3 and α5) conveys benzodiazepine (BDZ) sensitivity (Olsen and Sieghart, 2009; Rudolph and Knoflach, 2011; Rudolph and Mohler, 2014).

Modulation of GABA_A_R function by endogenous ligands may provide a physiologically and pathologically relevant mechanism to regulate GABA_A_R-associated functions and behaviour. In this respect, the positive allosteric actions of some endogenously occurring steroids have been identified to be of particular physiological and pharmacological significance over the course the past 3 decades. Specifically, following the pioneering discovery of the GABA_A_R potentiating actions of the synthetic anaesthetic steroid, Alphaxalone (5α-pregnan-3α-ol-11,20-dione Harrison and Simmonds, 1984) certain endogenous steroids, synthesised *de novo* in the brain and hence called neurosteroids (Baulieu, 1981) were shown to share this property. Such neurosteroids include the progesterone (PROG) metabolites 5α-pregnan-3α-tetrahydroprogesterone (5α3α-THPROG), 5β-pregnan-3α-tetrahydroprogesterone (5β3α-THPROG) and the deoxycorticosterone (DOC) metabolite 5α,3α-tetrahydrodeoxycorticosterone (5α3α-THDOC), which in common potently and stereo-selectively enhance GABA_A_R function in an allosteric fashion (Paul and Purdy, 1992; Belelli and Lambert, 2005). Intriguingly, the levels of such neurosteroids are rapidly elevated following acute stress (Purdy et al., 1991; Barbaccia et al., 2001; Morrow et al., 2009) and therefore, they may act to “fine-tune” the function of GABA_A_Rs and consequently influence HPA axis activity. In support, neurosteroids inhibit CRF release and exhibit anxiolytic and stress-protective properties (Crawley et al., 1986; Patchev et al., 1994, 1996; Carboni et al., 1996; Bitran et al., 1999).

Electrophysiological recordings have demonstrated that neurosteroids, such as 5α3α-THPROG and 5α3α-THDOC, potentiate the response of GABA (i.e. GABA-modulatory) at nanomolar aqueous concentrations, whilst at higher concentrations these endogenous regulators directly activate (i.e. GABA-mimetic) the GABA_A_R-channel complex (Callachan et al., 1987; Lambert et al., 1995; Shu et al., 2004). A significant body of evidence consistent with the presence of a specific neurosteroid binding site on the receptor has been provided during the past 25 years including: modulation of [^3^H] muscimol binding in solubilised preparations with minimal lipid content (Bureau and Olsen, 1993); clear enantioselectivity (Wittmer et al., 1996) and antagonism of both the *in vitro* and *in vivo* actions of neurosteroids by selective ligands i.e. 3α5α-17-phenylandrost-16-en-3-ol (17PA – Mennerick et al., 2004). A more definitive validation arose from site-directed mutagenesis studies, which revealed that neurosteroids interact with two distinct groups of amino acid residues located within the transmembrane (TM) domains of the GABA_A_R, which are both critical for their GABA-modulatory and the GABA-mimetic actions (Hosie et al., 2006). Subsequent reports have indicated that the neurosteroid binding pocket may possess a more complex structure than initially suggested with additional amino acid residues contributing to the modulatory actions of distinct, but structurally related steroid molecules (Akk et al., 2008; Chisari et al., 2010; Zorumski et al., 2013). Note that *in vitro* electrophysiological studies consistently report neuroactive steroids such as 5α3α to enhance GABA_A_R function at nM aqueous concentrations, suggesting the presence of a relatively high affinity binding site on the GABA_A_R protein. However, by virtue of their high lipid solubility, the actual concentration of neurosteroid achieved locally at the receptor protein will be in the micromolar range. Indeed, the differential accumulation of these steroids within the lipid membrane may serve to increase their local concentration, a suggestion which is in accord with a putative transmembrane docking site for neurosteroids. Such a scenario would enable and indeed facilitate neurosteroid access to a relatively low affinity binding site(s) located within the transmembrane spanning regions of the protein, *via* lateral diffusion through the membrane bilayer (Akk et al., 2005; Akk et al., 2007; Chisari et al., 2010).

A variety of factors have been shown to influence the apparent sensitivity of native GABA_A_Rs to neurosteroid modulation, including the subunit composition, phosphorylation state of the receptor and local steroid metabolism (Belelli and Lambert, 2005; Lambert et al., 2009; Nani et al., 2013). A detailed discussion of the relative contribution by each of these molecular mechanisms can be found in recent reviews (Herd et al., 2007; Lambert et al., 2009; Gunn et al., 2011).

Importantly, from a physiological perspective, naturally occurring plasma and brain levels of neurosteroids are estimated to be within a range required to potentiate GABA_A_R function. Further, the synthesis of these neuromodulators is dynamically regulated in response to physiological and pathophysiological challenges e.g. stress thus supporting the proposal for a significant role as endogenous regulators of GABA_A_R-mediated inhibitory transmission both in the central (CNS) and peripheral nervous system (PNS). In this review we will specifically appraise and focus on the documented and potential relevance of such actions for the early programming of the stress-neurocircuit and the regulation of stress-evoked responses. The potential pathological significance of such actions for stress-associated psychopathology will also be discussed.

## 2 Neurosteroids: endogenous modulators of GABA_A_R function

### 2.1 Neuronal and glia-mediated synthesis of neurosteroids

**Fig. 1:**
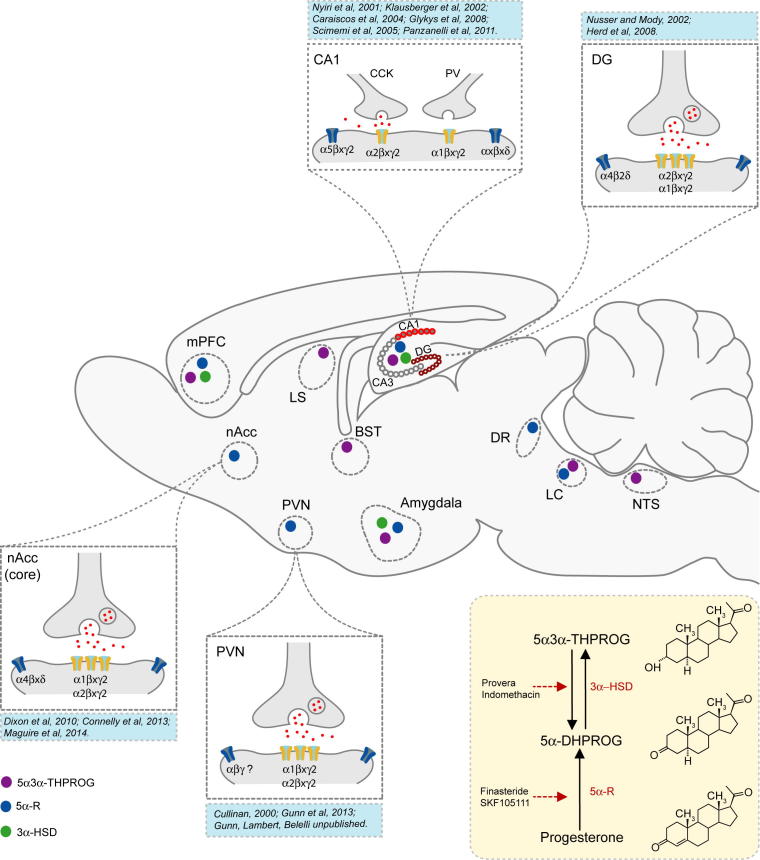
Synaptic and extra-synaptic GABA_A_R isoforms, neurosteroid synthesising enzymes and 5α3α-reduced pregnane steroids in the stress neurocircuitry. Diagrammatic representation of a sagittal section of a rodent brain depicting the expression profile of GABA_A_ receptor isoforms, the neurosteroid synthesising enzymes 5α-reductase (5α-R) and 3α-HSD and the distribution of GABA_A_R-modulating 5α3α-reduced pregnane steroids (e.g. 5α3α-THPROG), within the stress neurocircuitry. The reported expression of GABA_A_R subtypes is derived from both immunohistochemical and electrophysiological studies as referenced below. Note that GABA_A_R isoforms responsible for mediating phasic and tonic inhibition are expressed in a neurone-specific manner throughout the stress neurocircuitry. Note that for simplicity GABA_A_R isoforms expressed in various regions of the amygdala are not displayed but a discussion can be found in (Marowsky et al., 2012; Herman et al., 2013). Similarly, 5α-R, 3-HSD and 5α3α-reduced pregnane steroids have been detected across the stress axis albeit their levels exhibit distinct region-selective expression profile. References for the region-selective expression for the above enzymes are the following: prefrontal cortex, (PFC; Grobin et al., 2003; Castelli et al., 2013; Mitev et al., 2003); hippocampus, (Saalmann et al., 2007; Agis-Balboa et al., 2006; Castelli et al., 2013) and amygdaloid nuclei (Saalmann et al., 2007; Castelli et al., 2013; Do Rego et al., 2009; Mitev et al., 2003). The nucleus of the solitary tract (NTS) and the locus coreleus (LC), which directly innervate the PVN both exhibited immuno-reactivity for the 5α3α-reduced pregnane steroids (Saalmann et al., 2007), while 5α-R has been reported in LC and in the dorsal raphe (DR) nucleus (Castelli et al., 2013). Both the lateral septum (LS) and the bed nucleus of the solitary tract (BST) show immuno-reactivity for 5α3α-reduced pregnane steroid, while the relative expression of 5α-R and 3α-HSD remains to be determined. 5α-R expression has been shown in the nucleus accumbens shell, and to lesser extent in the core, while the presence of 3α-HSD and 5α3α-reduced neurosteroids remains to be determined. The hypothalamus displays an abundant expression of 5α-R and 3α-HSD abundantly (Li et al., 1997; Gao et al., 2002; Eechaute et al., 1999), although the expression in specific sub-regions requires further analysis. Within the PVN specifically, only the expression of 5α-R has been confirmed (Castelli et al., 2013), while dense 5α3α-reduced pregnane steroid immunoreactivity has been found in the periventricular zone (Saalmann et al., 2007). See also Section 2.1 and Fig. 2 for additional details. The bottom right panel depicts enzymatic steps that mediate 5α3α-THPROG synthesis from progesterone and describes the inhibitors of the individual enzymatic steps. (See above-mentioned references for further information.)

Although a significant proportion of neurosteroids are derived from peripheral sources, such as the adrenal cortex and ovaries (Paul and Purdy, 1992), the brain itself is a steroidogenic organ that is capable of the *de novo* synthesis of these neuromodulators (Purdy et al., 1991; Reddy, 2003; Barbaccia, 2004). Neurosteroids are synthesised from cholesterol *via* a series of steps that include the translocation of cholesterol across the mitochondrial membrane by the steroidogenic acute regulatory protein (StAR) and translocator protein 18 kDa (TSPO; formerly the mitochondrial peripheral BDZ receptor), the rate limiting step of steroid and neurosteroid synthesis. Within the mitochondria, cholesterol is converted to pregnenolone (PREG) by the P450 side-chain cleavage enzyme, CYP11A1 and then trafficked to the cytoplasm where it can be converted to a number of neurosteroids known to be active at the GABA_A_R, including 5α3α-THPROG. PREG is converted to 5α3α-THPROG following three sequential reactions catalysed by 3β-hydroxysteroid dehydrogenase (3β-HSD), 5α-reductase (5α-R) and 3α-hydroxysteroid dehydrogenase (3α-HSD), with progesterone and 5α-dihydroprogesterone (5α-DHP) being the respective intermediates (Do Rego et al., 2009). Two of these enzymes, 5α-R and 3α-HSD, are also involved in the conversion of the peripherally derived glucocorticoid metabolite, deoxycorticosterone (DOC) into 5α3α-THDOC (Fig. 1 – Karavolas and Hodges, 1990). Although a detailed discussion is beyond the scope of this review (but see Do Rego et al., 2009), two isoforms of 5α-R (type I and II) exist. While type I is the most abundant in both human and rodent brain, type II can be hormonally (e.g. testosterone) induced (Torres and Ortega, 2003).

A variety of brain cells have been shown to synthesise neurosteroids. Early studies described a role for astrocytes and glia in neurosteroidogenesis (Melcangi et al., 1993; Mellon and Deschepper, 1993). However, more recent immunohistochemical studies have suggested that the synthetic machinery necessary for neurosteroidogenesis, including StAR (King et al., 2002), CYP11A1 (Kimoto et al., 2001), 3α-HSD and 5α-R (Agis-Balboa et al., 2006), is highly expressed in excitatory principal cells in a number of brain regions, including the hippocampus and cortex (see Fig. 1 for details). Furthermore, immunohistochemical analysis of 5α3α-reduced pregnane steroid (e.g. 5α3α-THPROG, 5α3α-THDOC) localization within the brain, has detected the expression only in the cell bodies and dendrites of excitatory (glutamatergic) and, to a lesser extent, inhibitory (GABAergic) neurones in a brain region specific manner (e.g. Saalmann et al., 2007). Such observations are consistent with the proposed paracrine or indeed autocrine role of neurosteroids to locally regulate GABA_A_R-mediated inhibition (Agis-Balboa et al., 2006; Herd et al., 2007; Gunn et al., 2011). Although there is significant evidence to suggest a neuronal synthesis of neurosteroids, it is noteworthy that TSPO, the rate limiting step in steroid and neurosteroid synthesis (Rupprecht et al., 2010 – see Section 2.2), is highly expressed in glia, microglia and reactive astrocytes (Gavish et al., 1999; Kuhlmann and Guilarte, 2000; Casellas et al., 2002; Maeda et al., 2007) with only limited neuronal expression (Anholt et al., 1984; Bolger et al., 1984). Thus, it seems highly likely that, as originally postulated (Melcangi et al., 1993), glia are involved, at least to a degree, in the *de novo* synthesis of neurosteroids from cholesterol.

### 2.2 Local neurosteroid regulation of GABA_A_R function: physiological and pharmacological relevance

Recent experimental evidence provides considerable support to the notion that locally produced neurosteroids can enhance GABAergic transmission, and hence modulate neuronal excitability. Typically, the effect of these neuromodulators at synaptic and extrasynaptic GABA_A_Rs is manifest as a prolongation of the decay time of inhibitory post-synaptic currents (IPSCs) and as an increase in the tonic conductance respectively. Pharmacological manipulation of brain steroidogenesis has proved a useful strategy in elucidating the influence of local neurosteroid production upon shaping inhibitory transmission in specific neuronal populations. Thus, inhibition of 5α-reductase activity with the antagonist SKF105111 (11β-17-[bis(1 methylethyl) amino carbonyl – Fig. 1], resulted in a reduction in the decay time constant of spontaneous inhibitory post-synaptic currents (sIPSCs) recorded from neocortical pyramidal cells derived from the SKF-treated *cf* saline treated mice, indicating the presence of an endogenous neurosteroid tone in these neurones (Puia et al., 2003). In the hippocampus, miniature IPSCs (mIPSCs) recorded from dentate gyrus granule cells (DGGCs) were more sensitive to prolongation by the metabolically stable synthetic steroid analogue, ganaxalone (3β-methyl-5α3α-THPROG) in comparison to 5α3α-THPROG, raising the possibility that neurosteroid metabolism may be responsible for this differential sensitivity (Belelli and Herd, 2003). In particular, 3α-HSD, unlike 5α-reductase, is involved in both the synthesis and degradation of 5α3α-THPROG (and 5α3α-THDOC) as the cytosolic isoform catalyses the reductive formation of 5α3α-THPROG from 5α-dihydroprogesterone (5α-DHP), whilst the membrane bound isoform promotes the reverse, oxidative reaction (Li et al., 1997 – Fig. 1). Interestingly, in the dentate gyrus (DG) the membrane-bound isoform is prevalent and the inhibition of this enzyme with indomethacin, or methoxyprogesterone acetate (Provera – Fig. 1) resulted in a modest increase in both the mIPSC decay time constant and the bicuculline-sensitive tonic conductance, whereas CA1 GABA_A_R-mediated transmission remained unaffected by similar manipulations Furthermore, the treatment of brain slices with indomethacin or Provera alone enhanced the sensitivity of DGGCs to exogenously applied 5α3α-THPROG (100 nM, Belelli and Herd, 2003). Collectively, these observations indicate that the relative activity of 3α-HSD isoforms can influence the concentration of neurosteroids GABA_A_Rs experience and hence, may play a crucial role in shaping inhibitory transmission in a neurone, or even synapse, specific manner.

As described above, the translocator protein 18 kDa (TSPO) is located on the outer mitochondrial membrane, and in conjunction with the steroidogenic acute regulator protein (StAR, Sierra et al.*,* 2004) mediates the translocation of cholesterol across the outer mitochondrial membrane, the initial, and rate limiting step of steroid and neurosteroid synthesis (Rupprecht et al., 2010). Thus, provided a neurone contains the full complement of functionally active steroidogenic enzymes required for the *de novo* synthesis of 3α-reduced neurosteroids, then the stimulation of TSPO-mediated cholesterol translocation should increase the level of neurosteroids. Indeed, etifoxine is an anxiolytic drug that not only acts as a positive allosteric modulator of GABA_A_R function, but also stimulates TSPO to increase the cerebral production of 5α3α-THPROG, an effect that contributes to the behavioural actions of this drug (Verleye et al., 2005). More recent studies, in neurones from the mouse medial prefrontal cortex (mPFC), demonstrated that the selective TSPO ligand XBD173 (N-benzyl-N-ethyl-2-[7,8-dihydro-7-methyl-8-oxo-2-phenyl-9H-purin-9-yl] acetamide) produces an enhancement of the amplitude and duration of evoked IPSCs (eIPSCs) and mIPSCs. These effects were blocked by the 5α-reductase inhibitor, finasteride (Rupprecht et al., 2009). In addition, XBD173 is effective in rodent behavioural tests predictive of anxiolytic action, and these actions are attenuated by the TSPO antagonist PK11195 1-(2-chlorophenyl)-N-methyl-N-(1-methylpropyl)-3-isoquinolinecarboxamide; Rupprecht et al., 2009]. Importantly, the anxiolytic actions of XBD173 extend to human subjects, adding further credence to the proposal that compounds selectively targeting TSPO may provide novel anxiolytic agents with a reduced side effect profile (Rupprecht et al., 2009, 2010; Nothdurfter et al., 2012).

The treatment of brain slices with neurosteroid precursors such as PROG and DHP, have proved useful in establishing the steroidogenic potential of specific neurones. For example, in hippocampal CA1 pyramidal neurones, the inhibition of 5α-reductase with finasteride had no effect upon the decay time course of mIPSCs. However, the bath application of the 5α-reductase substrate, PROG (1 μM) resulted in a slowly developing, and finasteride-sensitive prolongation of the mIPSC decay kinetics (Sanna et al., 2004). Such observations may suggest that although CA1 pyramidal neurones do not exhibit a constitutive neurosteroid tone, the enzymes required for neurosteroidogenesis (i.e., 5α-reductase, 3α-HSD) are functionally expressed within these neurones. However, PROG had no effect upon the hippocampal expression of the 3α-HSD gene in either gender thus suggesting the lack of a simple correlation with precursor substrate levels (Mitev et al., 2003). Whilst the specific molecular mechanisms regulating enzymes gene expression and function remain to be fully elucidated, of significance, a recent study in rodent hippocampal CA1 pyramidal neurones has revealed activation of NMDA receptors (NMDARs) to rapidly promote neurosteroid synthesis (Tokuda et al., 2011) *via* a signalling mechanism involving p38 mitogen-activated protein kinase (MAPK), neuronal nitric oxide synthase and calcineurin (Izumi et al., 2008). Thus, NMDARs may play a pivotal role in the regulation of neurosteroidogenesis, potentially linking initial neural excitation to enhancement of neuronal inhibition.

In addition to a physiological role as regulators of GABAergic transmission, neurosteroids have been implicated in the pharmacological actions of a range of structurally diverse drugs acting on the GABA_A_ receptors. Thus, several line of evidence have implicated neurosteroids in some of the electrophysiological and behavioural effects associated with ethanol administration (VanDoren et al., 2000; Khisti et al., 2003; Sanna et al., 2004; Morrow et al., 2006; Izumi et al., 2007; Boyd et al., 2010; Tokuda et al., 2011). For example, the acute application of ethanol to CA1 pyramidal neurones promoted synthesis of 5α3α-THPROG resulting in an augmented peak amplitude and a prolonged decay time course of the mIPSCs recorded from such neurones (Sanna et al., 2004). Furthermore, in rats, acute ethanol treatment completely blocked the induction of hippocampal long-term potentiation (LTP), an effect dependent upon NMDAR-induced neurosteroid synthesis and, in agreement, sensitive to inhibition by either finasteride or the NMDAR antagonist AP-5 (Izumi et al., 2007; Tokuda et al., 2011).

Similarly, the effects of certain clinically important benzodiazepines (BDZs) may be mediated by (i) binding directly to the GABA_A_R (at a site between the α and γ subunits) to allosterically and rapidly enhance receptor function and (ii) activating TSPO to produce a delayed indirect enhancement of receptor function by increasing neurosteroid production. The best characterised BDZs which exhibit potentially pharmacologically relevant affinity for TSPO and the GABA_A_R include diazepam and midazolam, whereas the potent GABA_A_R-acting BZD clonazepam is a weak TSPO ligand (McCauley et al., 1992; Kalk et al., 2013). Evidence that both molecular targets may contribute to the pharmacological actions of such ligands has been offered by various independent investigations. Thus, for example, midazolam, a BDZ used clinically to facilitate the induction of anaesthesia, increased neurosteroid levels in CA1 pyramidal neurones and prevented the induction of LTP, a molecular correlate of memory function, in an acute brain slice preparation (Tokuda et al., 2010). Implicating neurosteroids, inhibiting their synthesis with finasteride, or preventing the GABA-modulatory actions of neurosteroids with the selective antagonist 17PA (Mennerick et al., 2004) attenuated the midazolam-induced impairment of LTP, consistent with a role for neurosteroidogenesis in the documented amnestic actions of this BDZ (Tokuda et al., 2010). In agreement with these *in vitro* findings, finasteride pre-treatment 1 day prior to midazolam injection, completely abolished the effects of this BDZ upon contextual fear learning (Tokuda et al., 2010). Similarly, midazolam exhibited a clear anticonvulsant action in the i.v. PTZ threshold model, which was significantly attenuated by finasteride treatment (Dhir and Rogawski, 2012). In contrast, clonazepam, an anxiolytic and anticonvulsant BDZ, did not induce changes in hippocampal neurosteroid synthesis, and had no effect upon LTP induction. However, consistent with a role for neurosteroidogenesis in the attenuation of LTP induction, when administered with the TSPO agonist FGIN (2-[2-(4-fluorophenyl-1H-indol-3-yl), or together with exogenous 5α3α-THPROG, clonazepam induced similar LTP-impairing effects to midazolam (Tokuda et al., 2010). However, the clinical relevance of midazolam actions have been questioned by a recent PET study suggesting the TSPO occupancy by clinically relevant concentrations of midazolam to be too low to contribute to the BZD *in vivo* profile in humans (Kalk et al., 2013). Therefore some caution may need to be exercised when extrapolating findings from rodent studies to humans.

## 3 GABA_A_ receptors and neurosteroids in the stress response

### 3.1 The stress circuitry and a role for GABA_A_Rs in the stress response

**Fig. 2:**
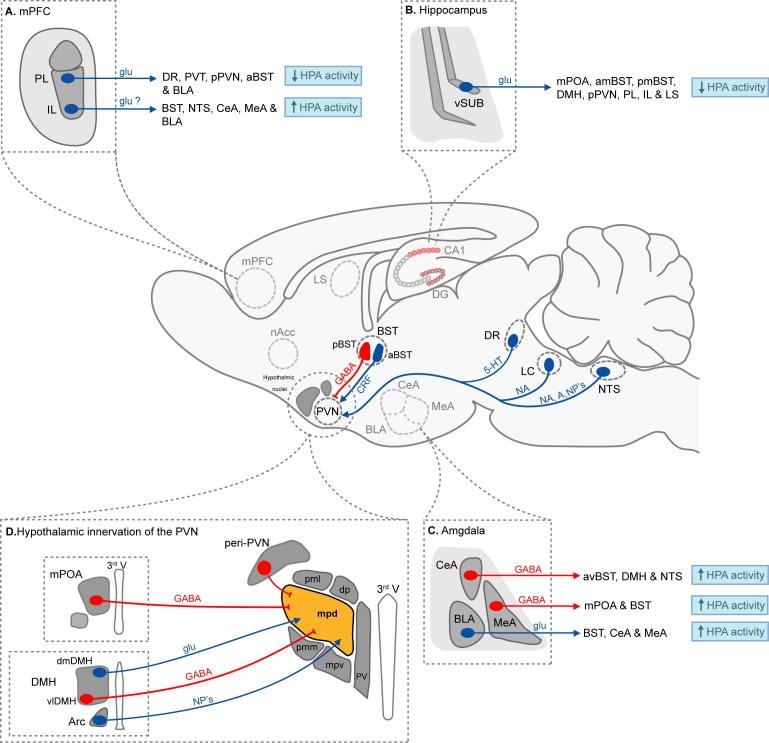
The stress neurocircuitry. A schematic representation of a sagittal section of a rodent brain, illustrating the neurocircuitry involved in the stress-induced regulation of HPA axis activity, with a particular emphasis on the nature of direct excitatory (blue) and inhibitory (red) projections to the PVN and the complex polysynaptic inputs mediating limbic and forebrain influences upon the stress response. The PVN receives direct noradrenergic, adrenergic and peptidergic innervation from the nucleus of solitary tract (NTS), as well as direct PVN-projecting noradrenergic and serotonergic afferents originating in the locus coreleus (LC) and dorsal raphe nucleus (DR). Projection from the bed nucleus of the stria terminalis (BST) are the main, non-hypothalamic forebrain inputs to the PVN, exerting both excitatory and inhibitory influences upon HPA axis activity. Thus, glutamatergic and CRFergic projections to the PVN originating in the anterior BST (aBST) stimulate HPA axis activity, whilst inhibitory GABAergic inputs originate in the posterior BST (pBST). Importantly the BST acts as primary relay site for limbic and forebrain inputs that have a substantial influence upon HPA axis activity (A, B and C). Direct inhibitory inputs to the PVN originate in a number of hypothalamic nuclei (D), including the medial preoptic area (mPOA), ventrolateral dorsal hypothalamus (vlDMH) and the peri-PVN (pPVN), while PVN-projecting afferents from the dorsomedial dorsal hypothalamus (dmDMH) provide intra-hypothalamic excitation to the HPA axis. Limbic and forebrain regions impart a significant influence upon the activity of the HPA axis, *via* indirect polysynaptic inputs to the PVN. The medial prefrontal cortex (mPFC) influences HPA axis activity in a subregion-specific manner (A). Thus, the prelimbic (PL) mPFC inhibits response to psychogenic stressors primarily *via* glutamatergic projections to inhibitory GABAergic relay nuclei (e.g. aBST, pPVN). In contrast, the infralimbic (IL) mPFC has been postulated to activate HPA axis activity possibly *via* projections to the NTS and/or CeA. Hippocampal stress output is mediated by glutamtergic projections from the ventral subiculum (vSUB) to primarily GABAergic relay nuclei (e.g. mPOA, pPVN, pBST and DMH), thereby inhibiting HPA axis activity (B). The amygdala exerts a primarily excitatory influence upon HPA axis activity, although this appears to occur in a subregion and stressor specific manner. Inhibitory, GABAergic projections from the central (CeA) and medial (MeA) amygdala disinhibit GABAergic relay nuclei (e.g. mPOA, aBST and DMH) to regulate responses to physical and psychogenic stressors respectively. Glutamatergic projections originating in the basolateral (BLA) amygdala innervate the CeA, MeA and BST, primarily increasing HPA axis activity in general in response to psychogenic stressors (C). The lateral septum (LS) also modulates HPA axis activity (pathways not shown) *via* polysynaptic inputs to the PVN. Furthermore, the nucleus accumbens (nAcc), which is primary component of the reward circuitry, may also be important in the regulation of HPA axis activity, providing a structural connection between the stress and reward circuitry. Although the influence the nAcc imparts upon HPA axis activity remains to be elucidated, projections from the nAcc core and shell to PVN-projecting brain regions such as the BST, lateral POA and lateral hypothalamus (LH) provide an anatomical basis for potential physiological effects upon the HPA axis (not shown). Further evidence for an interaction between the reward and stress systems is suggested by the observation that the receptors responsible for mediating the effects of glucocorticoids and CRF are expressed within the nAcc.

Maintaining physiological homeostasis in response to environmental and physiological challenges is essential for an organism to survive and such an ability to adapt is mediated through a number of tightly regulated and highly conserved interconnected systems. The hypothalamic paraventricular nucleus (PVN) is a brain region that is integral in the initiation of the neuroendocrine and autonomic response to a stressful challenge (Herman and Cullinan, 1997). Following stressor exposure, spinally projecting parvocellular neurones (located in dorsal and ventromedial regions) of the PVN rapidly modulate autonomic function, whilst neuroendocrine parvocellular neurones (located in dorsomedial regions), project to the median eminence and initiate activation of the HPA axis through the release of CRF (Ulrich-Lai and Herman, 2009). The HPA axis is a neuroendocrine pathway that couples the CNS to the periphery, and is regulated *via* hormonal feedback pathways (e.g. glucocorticoid, CRF) and a complex neurocircuitry comprised of mono- and polysynaptic pathways (Herman et al., 2003; de Kloet et al., 2005; Ulrich-Lai and Herman, 2009). As noted earlier, GABA has been identified as the dominant inhibitory neurotransmitter within the PVN (Decavel and Van den Pol, 1990) and, in agreement, exerts a significant inhibitory tone upon HPA axis function (Cullinan et al., 2008). Tract tracing studies in rodents have revealed the majority of the GABAergic afferents to the PVN to originate from a number of local hypothalamic nuclei (e.g. peri-PVN, dorsomedial hypothalamus, anterior hypothalamus, preoptic area (Roland and Sawchenko, 1993; Herman et al., 2002, 2003) as well as regions of the extended amygdala (e.g. bed nucleus of the stria terminalis, BST (Cullinan et al., 1993; Dong et al., 2001c; Dong and Swanson, 2004, 2006) – Fig. 2). Limbic and forebrain regions (e.g. ventral subiculum, medial prefrontal cortex and amygdala) also exert significant influence upon HPA axis activity, but do not directly innervate the PVN (Herman et al., 2003; Ulrich-Lai and Herman, 2009). Rather, these limbic and forebrain structures modulate HPA axis function *via* projections to a number of the local GABAergic nuclei, in particular regions of the BST (Dong et al., 2001b; Radley et al., 2009; Radley and Sawchenko, 2011 – Fig. 2) and peri-PVN (Herman et al., 2002), which act as neural hubs to integrate and relay limbic and forebrain influences upon HPA function to the PVN (Herman et al., 2002, 2003; Ulrich-Lai and Herman, 2009; Radley, 2012 – Fig. 2).

The GABAergic inhibitory tone is balanced by a comparable excitatory input to the PVN, which is heavily innervated by excitatory glutamatergic terminals (Decavel and van den Pol, 1992). Retrograde fluorogold labelling has revealed glutamatergic inputs to originate from regions of the ventromedial and dorsomedial hypothalamus (Ulrich-Lai et al., 2011) and to co-exist with local interneurons within the PVN (Boudaba et al., 1997; Herman et al., 2002), in contrast to GABAergic inputs, which derive exclusively from peri-PVN regions (Bali et al., 2005). In support, *in situ* hybridization studies have revealed within the PVN the expression of multiple ionotropic glutamate receptor subunits, particularly of the NMDA receptor family (Herman et al., 2000; Ziegler et al., 2005). In agreement, spontaneous action current firing of mpd neurones is abolished following the bath application of ionotropic glutamate receptor antagonists e.g. kynurenic acid, or AP-5 and DNQX (Zaki and Barrett-Jolley, 2002; Gunn et al., 2013) while intra-PVN microinjections of kynurenic acid significantly reduced glucocorticoid release following exposure to acute restraint stress (Ziegler and Herman, 2000). Collectively these findings support a primary role for glutamate in driving the excitability of these neurones.

Within the medial parvocellular region of the PVN, quantitative ultrastructural analysis has revealed that CRF-releasing neurones are the principle recipient of GABAergic inputs (Miklos and Kovacs, 2002) and dual *in situ* hybridization and immuohistochemical studies have demonstrated the expression of multiple GABA_A_R subunits (e.g. α1, α2, α3, α5, β1-3 and γ1, γ2) in the majority of these neurones (Cullinan, 2000; Hortnagl et al., 2013 – Fig. 1). In support, the GABA_A_R antagonist, bicuculline, increases the frequency of action current firing of rodent mpd CRF-releasing neurones (Hewitt et al., 2009; Gunn et al., 2013) and in agreement, plasma corticosterone levels increase, or decrease following the *in vivo* microinjection of bicuculline (Cullinan et al., 2008; Hewitt et al., 2009) and muscimol (Cullinan et al., 2008) respectively into the PVN.

The observations summarised above indicate that the balance between glutamatergic and inhibitory GABAergic transmission plays a fundamental role in regulating the output of the HPA axis. As a corollary to this suggestion, modulation of GABA_A_R function by endogenous neurosteroids may be important in “fine tuning” HPA axis function. The following section will review evidence in support of such a proposal and the relevance of these effects to the anxiolytic actions of neurosteroids.

### 3.2 Stress and neurosteroids

The anxiolytic actions of the endogenous neurosteroid 5α3α-THDOC were demonstrated almost 30 years ago (Crawley et al., 1986). However it was not until the pioneering studies by Bob Purdy in the early 1990s revealed that the brain levels of 5α3α-THDOC and 5α3α-THPROG were rapidly elevated in the rodent cortex and hypothalamus following exposure to acute swim stress that a putative role for neurosteroids as regulators of the stress response was postulated (Purdy et al., 1991). Of specific significance, adrenalectomy prevented the stress-induced increase in 5α3α-THDOC, but not 5α3α-THPROG, demonstrating the *de novo* brain synthesis of the latter neurosteroid (Purdy et al., 1991). Such observations suggested that neurosteroids synthesised *de novo* within the CNS and those derived from peripheral sources may be important modulators of the behavioural and/or neuroendocrine response to stress. Pertinent to such a hypothesis, the steroid synthesising enzymes, 5α-reductase and 3α-HSD are expressed within the hypothalamus (Li et al., 1997; Eechaute et al., 1999; Gao et al., 2002), while 5α3α-THPROG immunoreactivity can be detected within the extended amygdala and hypothalamus (Saalmann et al., 2007). Furthermore, several investigations have demonstrated similar increases in neurosteroid levels in response to a range of different stressors (Barbaccia et al., 1994, 1996), while the administration of 5α3α-THPROG and 5α3α-THDOC produce anxiolytic effects in various test of anxiety in rodents (Bitran et al., 1991; Wieland et al., 1991; Akwa et al., 1999). In further support, administration in rats of progesterone or agonists of TSPO induced increased levels of 5α3α-THPROG within the brain, which associated with anxiolytic-like effects that are attenuated by inhibitors of 5α-reductase or GABA_A_R antagonists (Bitran et al., 1995, 2000; Brot et al., 1997; Rupprecht et al., 2010).

The release of CRF from hypothalamic neuroendocrine parvocellular neurones triggers the activation of the HPA axis (Vale et al., 1981) and i.c.v. injection of this neuropeptide induces behaviours similar to those associated with stress and anxiety (Owens and Nemeroff, 1991). Interestingly, the anxiogenic effects of CRF in rats exposed to the elevated plus maze (EPM) can be prevented in a dose-dependent manner by pre-treatment with 5α3α-THPROG (Patchev et al., 1994). Furthermore, the pre-treatment of rats with 5α3α-THPROG, 5α3α-THDOC or PROG significantly attenuates the stress-induced increase in plasma ACTH and corticosterone (Owens et al., 1992; Patchev et al., 1996). Consistent with such *in vivo* actions, 5α3α-THPROG also suppresses the methoxamine [an α1-adrenoreceptor (α1-AR) agonist]-induced release of CRF in hypothalamic explant preparations, while having no effect upon basal release of this neuropeptide (Patchev et al., 1994). Such an effect is compatible with an action upon GABA_A_R as enhancement of the GABAergic tone similarly inhibits CRF release from parvocellular neurones (Owens et al., 1992). Congruent with such a proposal, in electrophysiological recordings made from neonatal (P18-26) mouse hypothalamus, we find that low concentrations (10–100 nM) of 5α3α-THPROG enhance GABA_A_R-mediated synaptic transmission of CRF-releasing mpd neurones of the PVN and inhibit their output (Belelli et al., 2009; Gunn et al., 2013). Furthermore, low concentrations of the DOC metabolite, 5α3α-THDOC, similarly reduces the output from neuroendocrine (Sarkar et al., 2011) and spinally projecting parvocellular neurones (Womack et al., 2006). The relevance of these studies in rodents to the regulation of the stress response in humans is emphasised by the demonstration that 5α-THDOC plasma levels exhibit a robust increase in response to a cholecystokinin-tetrapeptide (CCK-4)-induced panic attack in healthy volunteers. This increase is concomitant to stimulation of ACTH and cortisol release, both hallmarks of HPA axis activation, thus suggesting a specific possible role for 5α-THDOC in the termination of the stress/anxiety response following the CCK-4 challenge (Eser et al., 2005).

Collectively, the evidence gathered from molecular, immunohistochemical, electrophysiological and behavioural studies strongly supports the notion that endogenous 3α-reduced neurosteroids can reduce HPA axis activity by enhancing GABA_A_R function and such an action is consistent with their anxiolytic-like profile. Amongst the specific molecular targets mediating the actions of neurosteroids, the expression of α2-GABA_A_R in the PVN (see Section 3.1 and Fig. 1) may be of particular significance for their anxiolytic and stress-protective actions (Fig. 1). In agreement with this suggestion, in a recent report, “knock-in” mice, expressing a neurosteroid-insensitive α2 subunit (α2Q241M), exhibit an anxiogenic phenotype and an impairment of the anxiolytic response to injected neurosteroid, indicating a functional relevance (Durkin et al., 2011). Moreover, α2-containing GABA_A_Rs have been shown to mediate the anxiolytic action of classical BDZ’s, such as diazepam (Low et al., 2000) and are highly expressed not only in PVN, where the mRNA for the α2-subunit is highly co-localised with CRF mRNA signal (Cullinan, 2000), but also in other stress-sensitive brain regions including the amygdala, hippocampus and nucleus accumbens (Fritschy and Mohler, 1995; Hortnagl et al., 2013). Moreover, coupled with the inhibition of CRF, ACTH and corticosterone release by 5α3α-THPROG described above (Owens et al., 1992; Patchev et al., 1994, 1996), this finding is consistent with the suggestion that potentiation of α2-GABA_A_R isoforms may specifically contribute to the anxiolytic profile of neurosteroids.

Recently, the reported shift in the polarity of GABA actions (from hyperpolarising to depolarising) in neuroendocrine parvocellular neurones upon exposure to acute restraint stress (Hewitt et al., 2009) has led to the proposal that stress-induced modulation of GABAergic transmission by 5α3α-THDOC may actually facilitate rather than restrain activation of the HPA axis (Sarkar et al., 2011). Although such a scenario may be plausible, it does not appear compatible with their well-documented anxiolytic-like properties and the inhibitory actions of 5α3α-THPROG upon CRF, ACTH and corticosterone release as discussed above. Furthermore, the temporal profiles of the observed changes in GABAergic transmission and behavioural parameters on one side, and the stress-induced increase in neurosteroid levels on the other appear incongruent with a facilitatory effect of neurosteroid upon HPA axis activation. Thus, a decrease in GABAergic transmission and comparable changes in behavioural parameters related to GABAergic transmission occur rapidly (within ∼5 min) and with a similar time course following stressor exposure (Sanna et al., 1992; Barbaccia et al., 1996; Biggio et al., 2007). However, the levels of neurosteroids peak both in the whole brain and in the hypothalamus with a delay of ∼30–60 min after a stressful challenge (Purdy et al., 1991; Barbaccia et al., 2001), a time course mimicked by inducing stress by the direct injection of CRH, or ACTH (Torres et al., 2001). This temporal profile parallels the manifestation of their anxiolytic-like properties, a synchrony that appears incompatible with a facilitation of HPA axis activity.

In further albeit indirect support, the effect of stress on neurosteroid levels is mimicked by treatment with an anxiogenic GABA_A_R acting ligand but antagonized by anxiolytic benzodiazepines (Barbaccia et al., 1996; Biggio et al., 2007). Thus, neurosteroids appear to act in an adaptive fashion to curtail the extent and duration of the stress-induced inhibition of GABAergic transmission. Nevertheless, as noted above, other stress-sensitive regions implicated in the regulation of emotional states, e.g. hippocampus and amygdala, will contribute their anxiolytic properties (Bitran et al., 1999).

## 4 Programing of the stress neurocircuitry: a role for endogenous neurosteroids?

Throughout the lifespan, exposure to physiological and environmental challenges can have profound and enduring effects upon stress susceptibility and resilience (Lupien et al., 2009). However, in mammals, the pre- and post-natal periods appear particularly critical for brain development, and stressor exposure at this stage can severely alter physiological, behavioural and cognitive functions during adolescence and adulthood (McEwen, 2003; Bale et al., 2010; Franklin et al., 2012; Brunton et al., 2014). The following section will introduce how early postnatal experiences can modulate neurodevelopment with an emphasis on (1) the potential role for endogenous neurosteroids in the maturation of the HPA axis and overall stress neurocircuitry and (2) the impact of early-life adversity upon the stress-protective actions of endogenous neurosteroids. For a detailed discussion on the role of prenatal experience and specifically stress on programming of the HPA axis, see recent reviews Bale et al., 2010; Brunton and Russell, 2011; Brunton et al., 2014.

### 4.1 Early-life experience and HPA maturation

In rodents, the early postnatal period, is characterised by a marked hyporesponsivity of the HPA axis, occurring during days 4–14 of life (Sapolsky and Meaney, 1986). This ‘hyporesponsive’ period coincides with stages of axonal growth, synaptogenesis and myelination within key brain neurocircuits, and as such it has been postulated that the dampening of HPA axis reactivity, *via* a pituitary-mediated mechanisms involving GR inhibition of ACTH release (Schmidt, 2010), is an important mechanism for protecting the developing brain from excessive levels of glucocorticoids (Sapolsky and Meaney, 1986). Interestingly, alterations in the activation of limbic and forebrain GABAergic circuits have been implicated as potential candidates underlying reduced HPA function during the stress hyporesponsive period (Dent et al., 2007). The mother–pup interaction in rodents is crucial for the maturation of the HPA axis, and alterations in the level of maternal behaviours, such as licking-grooming (LG) and arched back nursing (ABN) can significantly influence the behavioural and neuroendocrine response to stress in adulthood (Liu et al., 1997; Francis and Meaney, 1999). Specifically, the adult offspring of high-compared to low-LG-ABN mothers exhibited decreased levels of hypothalamic CRF mRNA and increased hippocampal GR mRNA expression, which was accompanied by an increased sensitivity to glucocorticoid feedback and more modest HPA responses to acute stress (Liu et al., 1997). Interestingly, cross-fostering studies have revealed that these variations in maternal care may serve as a mechanism for the non-genomic transmission of individual differences in stress reactivity across generations e.g. *via* epigenetic mechanisms (Francis et al., 1999). The subsequent finding that alterations in hippocampal GR expression associated with differing levels of maternal care were caused by differences in DNA methylation of the GR gene promoter region and alterations in histone acetylation (Weaver et al., 2004) provided the first demonstration that maternal care can indeed modify the epigenomic state of stress-related genes. Moreover, these observations identified epigenetic modifications as candidate mechanisms to mediate gene-environment interactions that impact upon the maturation of the stress axis (Vialou et al., 2013).

Experimentally-induced manipulations to either enhance or impair maternal care have proved useful approaches to investigate the mechanisms underlying the neurobiological plasticity associated with early-life experience and maturation of the HPA axis (reviewed in Meaney, 2001; Franklin et al., 2012). For example, in rodents, postnatal handling comprising of short periods (i.e. 3–15 min) of daily separation of the pups from their mother during the first week(s) of life, promotes increased active maternal behaviours (i.e. LG, ABN) following the return of the pups to the home cage (Pryce et al., 2001; Fenoglio et al., 2006b). Similar to pups exposed to high levels of maternal care, early handling has been shown to result in decreased stress reactivity in adulthood, reduced hypothalamic CRF expression and increased hippocampal GR (Liu et al., 1997; Francis and Meaney, 1999; Sanchez et al., 2001). In contrast, adult offspring exposed to longer recurrent daily periods of maternal separation (e.g. 2–3 h) during the early postnatal period (e.g. P2-P14) displayed a significantly increased HPA response to acute stress, which was accompanied by a reduction in the expression of hippocampal GR mRNA and increased CRF mRNA in the hypothalamic PVN (Francis and Meaney, 1999; Meaney, 2001; Cirulli et al., 2003; Schmidt et al., 2004 although see Pryce et al., 2001; Lehmann et al., 2002). Interestingly, in a model of augmented maternal care, reprogramming of the stress response is mediated early-on (by P9) by a decreased expression of CRF in neurones of the PVN and only at later developmental stages by a reduced hormonal response to stressor exposure (by P23) and an epigenetically-induced enhancement of hippocampal GR expression (to increase the GR negative feedback), which takes place between P23-P45 (Avishai-Eliner et al., 2001; Korosi and Baram, 2009). Such observations suggest that plasticity of hypothalamic CRF-releasing neurones, resulting in the suppression of *crh* gene expression, is an early and potentially important process in the mechanism(s) responsible for mediating the effects of increased maternal care upon the stress response. Although not yet elucidated, it seems plausible that a similar temporal profile may be associated with the alterations in hypothalamic CRF, endocrine stress response and hippocampal GR expression that accompany negative early life experience (e.g. maternal separation, or fragmentation – see below).

### 4.2 Neurosteroids and HPA development

A potential physiological role for neurosteroids in the programming of the stress response was first proposed following the observation that administration of 5α3α-THPROG reduced the number of ultrasonic vocalisations (USVs), a measure of an anxious-like phenotype, in neonatal rat pups (P7) previously exposed to maternal separation (Zimmerberg et al., 1994, 1999). In support, in adult rats, the behavioural and neuroendocrine effects of repeated maternal separation early in life (P2-P10) were attenuated when 5α3α-THDOC was concomitantly administered during the separation period (Patchev et al., 1997). Consistent with a putative physiological role of neurosteroids early in life, the levels of 3α-HSD mRNA are elevated in the dentate gyrus of male and female rats at P7 (Mitev et al., 2003). Interestingly, the behavioural and neuroendocrine dysregulation associated with maternal separation in rats shows a gender specific profile as adult male, but not female rats displayed an anxious phenotype on the EPM following maternal separation (P5-P6; Mitev et al., 2003), although such effects may be additionally dependent upon the maternal separation protocol used (Zimmerberg and Kajunski, 2004). These sex-specific effects are likely to be a consequence of increased peripherally derived steroids (e.g. estrogen, progesterone) in females, as overectomised (OVX) female rats exposed to maternal separation exhibited an anxious-like phenotype on the EPM comparable to that observed in males. Interestingly, however, the concomitant administration of 5α3α-THPROG during maternal separation attenuated the behavioural and neuroendocrine consequences associated with postnatal stress in both genders (Mitev et al., 2003). Similar gender-specific regulation of neurosteroidogenesis has been described for the mPFC where restrain stress increased the mRNA and protein levels of 5α-reductase more significantly in male compared to female rats (Sanchez et al., 2009).

More recent studies have revealed that elevating the levels of neonatal (P5) 5α3α-THPROG altered exploratory and anxiety-like behaviours in adulthood (Modol et al., 2013), while reducing neurosteroid levels with the 5α-reductase inhibitor, finasteride produced an anxiogenic-like phenotype (Martin-Garcia et al., 2008).

The molecular mechanisms whereby neonatal levels of neurosteroids may regulate maturation of the HPA axis remain to be identified but the observation that during the first postnatal week activation of GABA_A_R mediates a depolarising response and thus may lead to secondary Ca^2+^ influx to trigger second-messenger-mediated changes in gene expression or protein function offers scope for future investigations (Fig. 4).

## 5 The effect of early-life experience upon neuroplasticity of the stress neurocircutry: a focus on GABA_A_R mediated inhibition and relevance to neurosteroid actions

### 5.1 Neuroplasticity of the PVN

The observation that a reduction in hypothalamic CRF expression precedes the reduced hormonal stress response and the epigenetically augmented expression of hippocampal GRs accompanying early handling (Avishai-Eliner et al., 2001; Korosi and Baram, 2009) suggests that plasticity of the hypothalamic CRF-releasing neurone is an early and important component of experience-induced programming of the stress response. Therefore, understanding the molecular and functional changes that occur in these neurones in response to variable maternal sensory inputs has been the focus of recent research. As described above (Section3.1) CRF-releasing neurones of the PVN receive excitatory glutamatergic, and inhibitory GABAergic inputs that signal *via* glutamate and GABA_A_ receptors respectively. Thus, recent studies have investigated whether augmented maternal care induces alterations in excitatory and inhibitory inputs to these neurones (Korosi et al., 2010). A combined approach of quantitative confocal microscopy, electron microscopy and electrophysiology, has revealed augmented maternal care to associate with a significant and selective reduction in the number of glutamatergic but not GABAergic synapses onto CRF neurones (Korosi et al., 2010). This neuroplasticity in excitatory transmission is accompanied by an increased expression of the transcriptional repressor neuron-restrictive silencing factor (NRSF), which negatively regulates *crh* gene transcription. Interestingly, although the reduced excitatory drive onto CRF-releasing neurones dissipated by adulthood, the elevated NRSF levels and suppression of CRF expression were preserved (Korosi et al., 2010), suggesting that reduced glutamatergic transmission in the developing PVN though crucial to initiate the reprogramming of CRF expression, is not necessary for its maintenance. In support of such a notion, recent studies using hypothalamic neuronal cultures have revealed that pharmacological blockade of ionotropic glutamate receptors is sufficient to down-regulate the expression of CRF mRNA (Karsten and Baram, 2013).

**Fig. 3:**
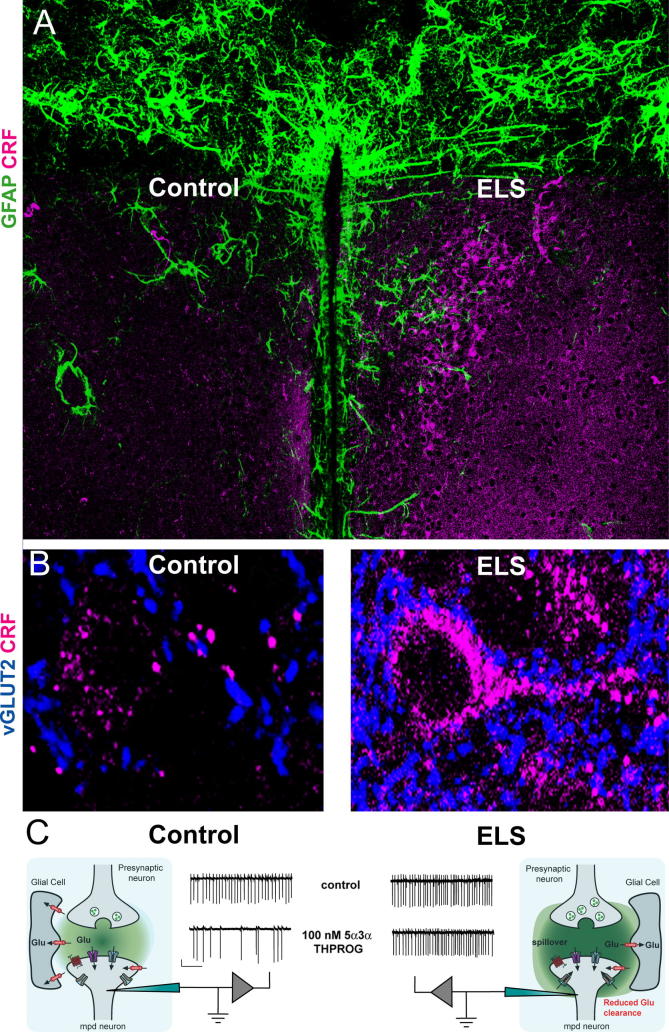
Adverse early-life experience induces significant functional and morphological plasticity in the neonatal mouse PVN. A. Immunohistochemical analysis illustrating the expression of CRF (pink) and the astrocytic cytoskeleton protein GFAP (green) in coronal sections of the neonatal hypothalamic PVN under control breeding conditions (left) and following exposure to ELS (right). Note that following ELS the expression of CRF in medial parvocellular neurones (mpd) increases while GFAP immunoreactive arbours become thinner. B. Immunuhistochemical analysis illustrating at high magnification the excitatory glutamatergic inputs measured by vGLUT2 expression (blue) apposing neonatal CRF-releasing neurones of mice bred under normal condition (left) or exposed to ELS (right). Note the significant increase in the number of excitatory glutamatergic (blue) inputs apposing neonatal CRF-releasing neurones exposed to ELS. C. Diagrammatic representation of the changes occurring at the glutamatergic synapses of CRF-releasing mpd neurones as a consequence of abnormal maternal care. Functional and molecular findings indicate an increase in phasic glutamatergic currents and an elevated excitatory tonic conductance in ELS compared to control neurones. The latter effect in mice exposed to ELS is mediated, at least in part, by the reduced clearance of glutamate from the synapse caused by a reduction in the expression of astrocytic glutamate transporters. In ELS-exposed mice, the increased glutamatergic drive associates with a loss of the inhibitory actions of the naturally occurring progesterone metabolite 5α3α THPROG upon the neuronal discharge of CRF-releasing mpd neurones. In this regard, compare cell-attached recordings before and after 5α3α THPROG shown to the right and left side of the diagrammatic synapses for Control and ELS neurones respectively. Note that the changes described above for ELS mice are shared by a genetic mouse model of postnatal depression, the δ “knock-out” mice, which also experience abnormal maternal care (Gunn et al., 2013).

More recently, we have explored whether an analogous albeit opposite form of neuroplasticity may take place in CRF-releasing neurones following a reverse manipulation i.e. exposure to adverse early-life experiences. Converging evidence from various laboratories has indicated a lack of consistent effects upon HPA programming by maternal separation protocols in mice (Millstein and Holmes, 2007; Own and Patel, 2013). Thus, we adopted a model of fragmented maternal care that produces enduring neuroendocrine and behavioural abnormalities (Rice et al., 2008). This experimental paradigm is based on a reduction of the nesting material (2/3) and the introduction of a fine gauge steel grid to house the dam and pups in order to prevent stable nest formation. Such an environment induces alterations in the maternal behaviour of the dam (i.e. shortened bout of nurturing behaviour and frequent shifts between behaviours) and recapitulates an important element of neglect in humans where the mother is present, yet the quality of care is impaired (Korosi and Baram, 2009; Baram et al., 2012). We have utilised a combined electrophysiological and immunohistochemical approach to investigate possible alterations in the inhibitory–excitatory balance impinging upon CRF-releasing neurones as a consequence of chronic ELS exposure in neonatal mice (P18-P26). Consistent with the working hypothesis, ELS induced a significant increase in the number and function of glutamatergic synapses apposing CRF-releasing neurones, whereas only modest plasticity was associated with the GABAergic inputs to these neurones (Gunn et al., 2013). Most prominently, a concomitant dramatic increase in the tonic glutamatergic conductance of CRF-releasing neurones was apparent for ELS-exposed mice and this up-regulation appeared to originate from impaired astrocytic function of the glutamate transporter (Gunn et al., 2013 – Fig. 3). Interestingly, the ELS-induced increase of the excitatory drive onto CRF neurones blunted inhibition of action potential firing in these neurones by physiologically relevant concentrations of 5α3α-THPROG (Fig. 3). This observation suggests that in response to a stressful challenge, the inhibitory actions of the stress-induced levels of neurosteroids (Purdy et al., 1991) may be compromised during adolescence and possibly in adulthood as a consequence of the plasticity induced by the negative early-life experience (Gunn et al., 2013). Although, only modest changes in GABA_A_R-mediated inhibition could be detected, a more detailed investigation is required to determine whether ELS exposure induces alterations in the expression of specific GABA_A_R isoforms or indeed the subcellular localisation of GABAergic inputs to mpd neurones (e.g. see Miklos and Kovacs, 2012).

Intriguingly, we have found that mice lacking the δ subunit of the GABA_A_R (δ^0/0^ mice) share with ELS mice similar PVN glutamatergic plasticity,namely an increased synaptic and extrasynaptic glutamatergic drive and a blunted neurosteroid action upon the neuronal discharge of CRF-releasing neurones (Gunn et al., 2013). These findings may appear paradoxical, as extrasynaptic δ-containing GABA_A_Rs, a putatively highly sensitive neurosteroid target (Belelli et al., 2002, 2009; Brown et al., 2002) are not expressed within the PVN (Wisden et al., 1992; Fritschy and Mohler, 1995, Hortnagl et al.*,* 2013; Gunn et al., 2013 although see Sarkar et al., 2011). However, a plausible explanation is offered by the observation that δ^0/0^ pups exhibit phenotypic features of abnormal maternal care (Maguire and Mody, 2008; Gunn et al., 2013), and in agreement, in common with ELS mice, display a significant and long lasting up-regulation of CRF expression within the PVN Furthermore, exposure to early-life adversity dramatically exacerbates the δ^0/0^ phenotype with greatly increased perinatal and adult mortality (Gunn et al., 2013). A reduced maternal care for δ^0/0^ pups may not be altogether unexpected given that an increased dopamine release from the ventral tegmental area (VTA) to the nucleus accumbens shell in the dam is associated with the onset, duration and magnitude of licking-grooming bouts, an important component of maternal care in rodents (Champagne et al., 2004; Russo and Nestler, 2013), and presynaptic δ-GABA_A_Rs may regulate the dopaminergic output of VTA neurones (Xiao et al., 2007; Vashchinkina et al., 2012). Further, intriguingly, a mouse model of chronic stress exhibits a long-lasting significant decrease in δ-GABA_A_R expression in the VTA (Warren et al., 2013). Nevertheless, additional studies, such as cross-fostering (Priebe et al., 2005), are required to establish the relative contribution genetic and environmental factors make to the described δ^0/0^ phenotype (Maguire and Mody, 2008; Gunn et al., 2013). In this regard, the reported association of SNPs in the GABRD gene with childhood-onset mood disorders raises the prospect that specific GABA_A_R genetic abnormalities may underlie susceptibility to stressful experiences and thus contribute to the development of psychopathology in humans (Feng et al., 2010).

Given the pivotal role of NRSF in the regulation of CRF gene expression in the mouse model of augmented maternal care described by Korosi et al. (2010), the contribution of NRSF, or indeed other transcription repressors, to the PVN plasticity in offspring of models of impaired maternal cares (e.g. of ELS and δ^0/0^) deserve a detailed investigation. Nevertheless, the evidence described is consistent with the notion that alterations in excitatory glutamatergic transmission coupled with blunted neurosteroid actions early in postnatal development may be an important molecular signal to initiate programming of the stress axis. Further, from a clinical perspective, abnormal regulation of glutamatergic transmission in animal models of chronic stress appear relevant and consistent with recent therapeutic strategies targeting inhibition of ionotropic glutamate receptors, e.g. NMDA receptors for the treatment of stress-related psychiatric disturbances e.g. depression (Niciu et al., 2014).

### 5.2 Neuroplasticity of extra-hypothalamic stress centres

As described in Section 3.1, the hypothalamic PVN sub-serves the combined role of integrating multiple inputs from hypothalamic and extra-hypothalamic stress-responsive regions and initiating the stress response. However, multiple forebrain and limbic structures, including the hippocampus, amygdala and mPFC appear to operate in a parallel fashion to process and integrate psychogenic and systemic stimuli to coordinate HPA axis activity appropriately. The network activity of these limbic regions is determined in a large part by the activity of inhibitory GABAergic interneurons (Klausberger and Somogyi, 2008; Ehrlich et al., 2009; Taniguchi, 2014), and as such, dysfunction in GABAergic transmission within these brain regions may be predicted to impart susceptibility to stressor exposure and to the development of psychiatric disorders. Indeed, there is substantial preclinical and clinical evidence indicating that alterations in GABAergic inhibition may be a contributing factor in the pathogenesis of affective disorders such as depression (see section below; Krystal et al., 2002; Sanacora et al., 2004; Luscher et al., 2011; Maciag et al., 2010). As noted above, we have found in mice that exposure to fragmented maternal care early in life results in limited apparent plasticity in the GABAergic inputs to PVN mpd neurones (although see below). However, there is substantial evidence that early-life experiences causes alterations in GABA_A_R-mediated signalling in other stress-sensitive brain regions. The following sections will discuss the influence of early-life experience upon the maturation of GABAergic circuits within these limbic and forebrain regions, highlighting the potential role for neurosteroids within this process and the implications that plasticity in these brain structures may have upon the actions of these endogenous modulators upon GABAergic transmission.

The quality of early-life experience has been associated with alterations in GABA_A_R subunit expression in a number of brain regions within the stress neurocircuitry. Moreover, such plasticity appears concomitant to perturbations in behavioural and neuroendocrine responses to stressor exposure in adulthood (Caldji et al., 1998, 2000, 2003; Hsu et al., 2003). Alterations in GABA_A_R expression in regions of the amygdala, NTS and locus coeruleus (LC) were inferred from autoradiography binding studies using [^3^H] flunitrazepam, where animals exposed to postnatal handling (P1–P14) displayed an increase in central BDZ binding compared to non-handled and maternally separated rats (Caldji et al., 2000). This increase in central BDZ binding in handled animals was accompanied by an increase in γ2 mRNA in these brain regions and reduced fearfulness in response to novelty (Caldji et al., 2000). Comparable perturbations, namely a significant reduction in the expression of the α1 subunit but up-regulation of α2 subunit levels, have been described for DGGCs of rat pups exposed to two periods of handling with maternal separation (HMS) on P9 (30 min) and P10 (360 min; Hsu et al., 2003). In general, adult offspring that received a high level of maternal care (i.e. high LG-ABN) displayed significantly greater mRNA encoding α1 and β3 subunits in limbic and forebrain regions when compared to those that received low levels of maternal care (Caldji et al., 2003). Intriguingly, alterations in GABA_A_R subunit expression associated with variations in maternal care are most pronounced in regions of the amygdaloid complex, an important brain region for the processing of “psychogenic” stressors (Ulrich-lai and Herman, 2009) and a key area implicated in the development of fear learning (Roozendaal et al., 2009). Specifically, the central (CeA), basolateral (BLA) and lateral (LA) amygdaloid nuclei of adult offspring that experienced high-levels of maternal care, displayed significantly greater mRNA expression levels of both γ1 and γ2 subunits, the latter being important in mediating the majority of actions by benzodiazepines. Conversely, the mRNA levels of α3 and the BZ-insensitive α4 subunit increased in the CeA and BLA of mice exposed to low quality maternal care (Caldji et al., 2003). Such changes will impact upon the functional and pharmacological properties of GABA_A_ receptors, including, potentially their sensitivity to neurosteroids. However, as noted above (Sections 1 and 2) in addition to the receptor subunit composition, the effects of neurosteroids are potentially, influenced by other mechanisms such as local steroid metabolism and phosphorylation state of the receptor or associated proteins (Herd et al., 2007). Thus, elucidating possible alterations in these regulatory processes e.g. altered expression of steroidoigenic enzymes, may warrant future detailed investigations. A precedent in this respect is offered by the demonstrated alteration in the activity of steroidogenic enzymes e.g. 5α-reductase in forebrain regions in a socially isolated mouse model of chronic stress (Dong et al., 2001a; Pibiri et al., 2008).

The amygdala has been identified as a key structure in mediating the anxiolytic (Akwa et al., 1999; Engin and Treit, 2007) and the antidepressant (Shirayama et al., 2011) effects of 5α3α-THPROG. Therefore, for this brain region understanding the impact of such alterations in GABA_A_R gene expression on synaptic and extrasynaptic inhibitory signalling may be particularly pertinent.

The ELS–induced alterations in GABA_A_R expression in the extra-hypothalamic regions described above are likely to occur in conjunction with alterations in excitatory glutamatergic transmission. Similarly to the GABAergic system, relatively little is known regarding the specific functional alterations in glutamatergic transmission at the synaptic level (i.e. properties of EPSCs) within limbic and forebrain structures following early life stress. However, perturbations in glutamatergic transmission have been inferred from morphological and functional studies, primarily in the hippocampus (Martisova et al., 2012). For example, in rats, exposure to fragmented early-life stress (as described for mice in Section 5.1) results in significant cognitive deficits in adulthood (Brunson et al., 2005; Ivy et al., 2010), which are associated with impaired hippocampal LTP, dendritic atrophy and a reduction in spine numbers (Brunson et al., 2005; Ivy et al., 2010; Wang et al., 2011 reviewed in Regev and Baram, 2014). Furthermore, in rats, offspring exposed to low levels of maternal care exhibited enhanced basal NMDAR function (measured by an increase in NMDA/AMPA evoked EPSCs ratio) in the DG, which was associated with increased NR1, NR2A and NR2B expression (Bagot et al., 2012 reviewed in Tse et al., 2012; Timmermans et al., 2013). Collectively these observations indicate alterations in glutamatergic transmission in stress-related brain regions are likely to accompany stress-induced plasticity in the GABAergic system during development. Elucidating the specific functional changes in excitatory/inhibitory balance both at the synaptic and extrasynaptic levels and establishing how plasticity in the GABAergic and glutamatergic systems may interact to influence network activity, and hence output from these extra-hypothalamic brain areas, should offer an important focused scope for future studies.

## 6 Stress-associated interneuron plasticity and neurosteroids

### 6.1 Dysfunction of the GABAergic system

Exposure to early-life stress has consistently been shown to induce significant plasticity in the functional and morphological properties of principal cells in limbic and forebrain regions (Fenoglio et al., 2006a; Lupien et al., 2009; McEwen and Morrison, 2013; Regev and Baram, 2014), However, the changes in the expression of GABA_A_R subunits in limbic and forebrain regions induced by early-life experience may also be accompanied by alterations in the number and/or localisation of GABAergic interneurons including those surrounding the PVN region (Bali et al., 2005). Such adaptations may have a significant impact upon the development of an organism’s ability to process and cope with stressor exposure. Indeed, GABAergic interneurons represent a highly diverse class of inhibitory neurones that interact with glutamatergic principal cells in a domain-specific manner, supporting the temporal dynamics of synaptic transmission and network oscillations, both of which are essential for implementing specific brain states (Markram et al., 2004; Klausberger and Somogyi, 2008; Lewis et al., 2012). Alterations to interneurone function albeit with some differences dependent on the species investigated and the protocol employed, have been inferred from immunohistochemical studies measuring the levels of calcium binding proteins [CBPs e.g. parvalbumin (PV), calretinin, calbindin] expressed primarily in GABAergic interneurons. Thus, a number of rodent brain regions including the prelimbic prefrontal cortex (plPFC) (Brenhouse and Andersen, 2011), anterior cingulate cortex (ACd), precentral medial cortex (PrCm; Helmeke et al., 2008) and the dentate gyrus (Seidel et al., 2008) exhibit reduced levels of CBPs following various manipulations of maternal care. Although these studies do not provide direct functional evidence supporting ELS-induced alterations in GABAergic transmission, they do suggest potential plasticity of inhibitory interneurons following ELS, as the reduced PV expression is indicative either of a decrease in interneuron numbers, and/or alterations in interneuron activity. A precedent in this regard is offered by the documented association between abnormal cortical levels of PV and GABAergic dysfunction both in animal models of schizophrenia and schizophrenic patients (Lewis et al., 2012). Furthermore, clinical studies also highlight a loss of GABAergic interneurons in patients suffering from stress-related psychopatholgy e.g., depression (Maciag et al., 2010) and a loss of GABAergic signals is apparent in imaging studies (Sanacora et al., 1999).

Different types of inhibitory interneurons synapse onto distinct subcellular regions of principle cells (e.g. axon initial segment, dendrites, cell soma), thereby imparting distinct spatiotemporal GABAergic conductances onto the pyramidal cell, the pattern of which changes during particular physiological and pathophysiological states (Lewis et al., 2012; Takesian and Hensch, 2013). Both in the hippocampus and cortex, current evidence supports a selective targeting of interneuron subtype to principal neurones cellular domains associated with the expression of a distinct GABA_A_R subunit complement (Mohler, 2012; Fritschy and Panzanelli, 2014). Thus, experience-induced alterations in dendritic/axonal morphology, associated changes to the subcellular localisation of GABAergic interneuron synapses onto principle cells, or functional plasticity in the GABA_A_Rs expressed at these distinct synapses, may significantly influence the neuronal and behavioural response to stress in adulthood. In support of such a notion, the propagation of locally evoked activity in the DG relative to that evoked the in CA1 was reduced in a rodent model (chronic mild stress [CMS]) of depression. Importantly such a reduction in activity propagation in the DG relative to the CA1 was associated with the behavioural phenotype of CMS-exposed animals in the forced swim (FST) and open field tests (OFT). These findings are consistent with the proposal that alterations in network dynamics may underlie behavioural changes accompanying chronic stress (Airan et al., 2007).

### 6.2 Stress-associated plasticity: a role for neurosteroids?

**Fig. 4:**
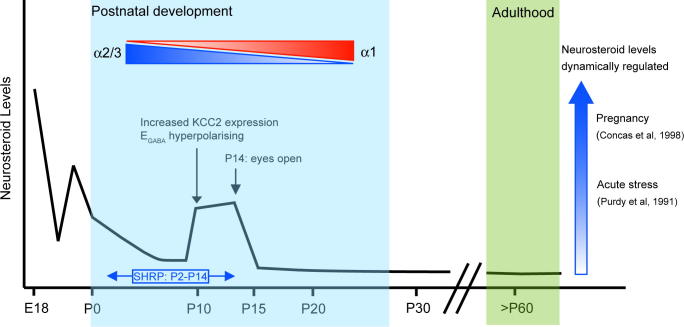
The brain levels of 5α3α-THPROG are developmentally regulated. Diagrammatic illustration of rat cortical levels (arbitrary units) of 5α3α-THPROG across various developmental stages. During embryonic development the levels of 5α3α-THPROG decrease considerably at E20 before increasing prior to parturition. The relatively high levels of 5α3α-THPROG at birth (P0), decline gradually over the first postnatal week until P10, when they rise again to levels comparable to those observed at birth. This transient increase lasts until P15, when the levels of 5α3α-THPROG decline back to basal values. Interestingly, the early postnatal period, which is associated with generally elevated levels of 5α3α-THPROG coincides with the so-called stress hypo-responsive period (SHRP, P2-P14). Furthermore, the secondary transient peak (at P10) in cortical neurosteroid levels coincides with significant developmental changes associated with GABA_A_R-mediated inhibition within the CNS. As detailed, during this developmental stage, at P9–P10 the actions of GABA switch from being depolarising to hyperpolarising as a consequence of the increased expression of the potassium-chloride co-transporter, KCC2, thus causing a subsequent reduction in intracellular Cl-concentration. Shortly after this change, the further development of the rodent visual cortex begins at ∼P14 following eye opening. This early period of significant neuronal and network maturation is additionally accompanied by significant developmental changes in the GABA_A_R subunit expression, typified by a switch from the embryonic α2/3 to adult α1 subunits, which takes places throughout the second and third postnatal weeks. Although, in adulthood the basal levels of 5α3α-THPROG are typically lower than during early postnatal development, they are dynamically elevated in response to specific physiological conditions, including acute stressor exposure and during pregnancy.

The early postnatal period, which is characterised by the stress-hyporesponsive period (SHRP, P2–P14 – Fig. 4), represents a time of considerable plasticity within the CNS with ongoing neuronal migration, synaptogenesis and apoptosis. Interestingly, in rodents the cortical levels of the endogenous neurosteroid, 5α3α-THPROG vary considerably during postnatal development, falling precipitously prior to parturition and remaining relatively lower for the first week of life (Grobin and Morrow, 2001 – Fig. 4) . However, during the second postnatal week, a transient increase in the levels of 5α3α-THPROG between P10–P14 is observed, which return to low adult levels on P15 (Grobin and Morrow, 2001 – Fig. 4). This dynamic regulation of neurosteroid levels during early developmental stages raises the prospect of a likely physiological role for neurosteroids in cortical maturation. Consistent with such a notion, perturbations in the levels of endogenous neurosteroids, such as 5α3α-THPROG during early postnatal development have a significant impact upon the localisation of PV-expressing GABAergic interneurons of the PFC in adulthood (Grobin et al., 2003). Specifically, the ratio of PV-expressing neurones in the deep (layers V–VI) vs superficial (layers I–III) layers of adult PFC is increased two fold in rats following neonatal (P1 or P5) administration of 5α3α-THPROG (10 mg/kg – Grobin et al., 2003). An enhancement of GABA_A_R function by the neurosteroid appears to be the likely mechanism underlying the observed anatomical rearrangement as similar changes in interneuron placement become apparent following postnatal exposure to the benzodiazepine, flunitrazepam (Grobin et al., 2004). Such an effect of 5α3α-THPROG upon interneuron localisation is consistent with the observation that ambient GABA promotes cortical entry of tangentially migrating neurons derived from the medial ganglionic eminence (MGE). Similarly, a BZ (diazepam)-mediated enhancement of GABA_A_R function increased the migration of MGE neurones (Cuzon et al., 2006). Interestingly, prenatal exposure to ethanol also promotes premature tangential migration of GABAergic interneurons (a proportion of which originate in the MGE) by increasing the ambient levels of GABA and enhancing the sensitivity of migrating interneurons to this neurotransmitter (Cuzon et al., 2008). Given the documented effects of both BZs and ethanol upon hippocampal neurosteroidgeneis (Morrow et al., 2006, 2009; Tokuda et al., 2010, 2011; see Section 2.2), it is tempting to speculate that at least some of these effects may be indeed mediated by endogenous neurosteroids (Morrow et al., 2006, 2009; Tokuda et al., 2010, 2011). Nevertheless it should be noted that dynamic alterations in GABA_A_R subunit expression have also been implicated in the process of interneuron migration (Cuzon Carlson and Yeh, 2011) and thus, may provide an additional potential direct or indirect e.g. *via* neurosteroids mechanism for the enhanced GABA sensitivity following ethanol exposure. Moreover, intriguingly, the secondary early postnatal neurosteroid peak at P10 in cortex coincides with the switch in the polarity of GABA actions from depolarising to hyperpolarising (due to the increased expression of the potassium-chloride co-transporter, KCC2 and subsequent reduction in intracellular Cl-concentration – Fig. 4) suggesting a possible mechanistic link between the two phenomena.

A much improved understanding of the impact of early life adversity upon the integration and localisation of interneurons into principle cell networks and the timing of the switch in the polarity of the GABA response offers ample and immediate scope for future investigations. As disruption of maternal care alters hippocampal neurosteroid levels (Kehoe et al., 2000; Frye et al., 2006), it is reasonable to predict that changes in neurosteroidogenesis may occur in a number of brain regions and thus, may be a more widespread important mechanism contributing to early-life stress-induced neuronal plasticity than is currently appreciated.

## 7 Neurosteroid action, GABA_A_Rs and stress: implications for stress-related psychopathology

It is widely accepted that stress represents a significant risk factor for the development of psychiatric disturbances e.g. depression (Lupien et al., 2009; Heim et al., 2010; Sickmann et al., 2014). Despite the prevalence of major depressive disorders as a leading cause of disability worldwide, the neurobiological processes that underlie the emotional, cognitive and neuroendocrine perturbations associated with these multifactorial disorders remains relatively poorly understood, hindering the development of novel therapeutics. Specifically, the most commonly prescribed antidepressants, the selective serotonin reuptake inhibitors (e.g. fluoxetine) have a relatively long latency for therapeutic benefit (2–3 weeks) and are only moderately effective, with more than one third of depressed patients remaining treatment-resistant (Racagni and Popoli, 2008). Over recent years significant efforts to identify novel pathogenic and therapeutically relevant mechanisms for major depressive disorders have gathered convincing evidence from both preclinical and clinical studies implicating both GABAergic and glutamatergic systems (Sanacora et al., 1999, 2012; Krystal et al., 2002; Luscher et al., 2011; Popoli et al., 2012). Such findings are consistent with (i) a prominent role played by the excitatory-inhibitory balance in the regulation of the stress response, and (ii) the documented association between chronic stress exposure with consequent dysfunctional regulation of the stress response and the subsequent development of psychopathology (Lupien et al., 2009; Franklin et al., 2012). The following section will review this field of research with a specific emphasis on the putative pathological and potentially therapeutically relevant roles of GABA and neurosteroids to alter the inhibitory-excitatory balance. A detailed review of the glutamatergic hypothesis of depression is beyond the scope of this review and can be found elsewhere (see Sanacora et al., 2012; Popoli et al., 2012).

### 7.1 GABA, neurosteroids and depressive disorders

Findings form clinical and preclinical studies have provided compelling evidence for an association between alterations in GABAergic transmission and stress-related psychiatric disorders including depression (Sanacora et al., 2000; Krystal et al., 2002; Luscher et al., 2011; Mohler, 2012; Zorumski et al., 2013). Abnormal GABA signalling in major depressive disorders was initially suggested following studies from the early 1980s that demonstrated reduced plasma and cerebrospinal fluid (CSF) levels of GABA in patients suffering from depressive disorders (Gold et al., 1980; Petty and Schlesser, 1981), an observation subsequently confirmed by magnetic resonance spectroscopy studies of GABA levels (Sanacora et al., 1999, 2004; Hasler et al., 2007). Moreover, a reduction in the density of GABAergic interneurons has been reported in the cortex and amygdala of depressed patients (Rajkowska et al., 2007; Maciag et al., 2010; Guilloux et al., 2012). Post-mortem microarray studies have additionally indicated widespread alterations in the expression of GABA_A_R subunits and/or GABA_A_R-associated binding proteins specifically in suicide victims suffering from major depressive disorders (Sequeira et al., 2009), thus reinforcing the notion of a pathologically relevant association between GABAergic deficits and depression.

Although there are considerable limitations in interpreting behavioural data from rodent models of psychiatric disorders (Nestler and Hyman, 2010), evidence emerging from such studies is nevertheless consistent with a deficit in GABAergic inhibition in the pathogenesis of depressive-like behaviours. For example, heterozygous deletion of the γ2 GABA_A_R (γ2^+/−^) subunit results in a brain region-specific reduction (6–35%) in [^3^H] flumazenil binding and receptor clustering, with the most pronounced changes occurring in limbic and cortical regions (Crestani et al., 1999). Importantly, this deficit in GABA_A_R function associates with increased behavioural responses to aversive stimuli such as novelty, exposed space and brightly illuminated areas, all indicative of an anxious-like phenotype (Crestani et al., 1999). Recent studies have revealed that selectively inducing the heterozygous γ2 subunit deletion in developing forebrain glutamatergic neurones not only recapitulates the behavioural deficits associated with the global γ2^+/−^ (Earnheart et al., 2007), but also results in an increased basal HPA axis activity (Shen et al., 2010). Although currently not known, it is tempting to speculate that neurosteroids, the levels of which are normally elevated during early development (Fig. 4) may be implicated in the development of these perturbations. The observation that the development of a hyperactive HPA axis during the neonatal period associates with an anxious and depressive adult phenotype when a genetically-engineered γ2-GABA_A_R deficit is introduced embryonically, but not in the fourth postnatal week, is intriguing in this regard. Interestingly, however, pharmacological manipulation of GABA_A_R function at specific developmental time points with the BDZ, diazepam, associated with the development of distinct behavioural phenotypes in adulthood. Thus, diazepam administration at P10–P16 (but not during subsequent weeks) resulted in increased anxiety-like behaviour on the elevated plus maze (EPM) in adulthood, while the same treatment selectively between P29-35 resulted in an increased immobility i.e. a possible surrogate of depressive-like behaviour in the FST in adulthood (Shen et al., 2012). These observations suggest that the generation of anxiety- and depressive-like behaviours as measured in the EPM and FS tests respectively, are highly sensitive to manipulation of GABAergic inhibition mediated by γ2-GABA_A_Rs during distinct critical periods of postnatal development (Shen et al., 2012). Whether neurosteroids contribute to these phenotypes is not known but warrants further investigation, particularly in the light of the role of neurosteroidogenesis in the midazolam mediated effects in hippocampal function and the documented agonist activity of diazepam at the TSPO receptor (Tokuda et al., 2010; McCauley et al., 1992).

Support for 3α-reduced neurosteroids in the pathogenesis of depressive disorders has derived from preclinical and clinical studies of the past two decades, which postulated a therapeutically relevant contribution by these endogenous modulators to the anxiolytic properties of some SSRIs (e.g. fluoxetine – (Guidotti and Costa, 1998; Eser et al., 2006; Uzunova et al., 2006; Schule et al., 2014). Thus, the plasma and CSF levels of 5α3α-THPROG are reduced in depressed patients (Romeo et al., 1998; Uzunova et al., 1998) and, similarly in animal models of depression, or post-traumatic stress disorder (PTSD) in a brain region specific manner (Serra et al., 2001, 2008; Pinna, 2010). Furthermore, exogenous neurosteroid treatment produces antidepressant-like effects in a rodent model of chronic social isolation (Pibiri et al., 2008; Pinna*,* 2010; Evans et al., 2012). Of note and potential clinical relevance, the levels of 3β-reduced neurosteroids e.g. isopregnanolone (3β-THPROG), which at relatively high concentrations is a negative modulator of GABA_A_R function (Maitra and Reynolds, 1998), appeared increased in chronic fatigue syndrome, a condition sharing features with depression (Murphy et al., 2004). Similarly, a chemically-induced panic attack was associated with a pronounced increase in the levels of isopregnanolone in panic disorder patients, who in absence of panic attacks display decreased levels of the same neurosteroid relative to a cohort of healthy controls (Strohle et al., 2002, 2003). Thus, collectively, these findings suggest a potential contribution of this steroid to the symptomatology of psychiatric disorders. Interestingly, studies in rodents have additionally demonstrated that the brain levels of 5α3α-THPROG are normalised following acute treatment with the SSRI, fluoxetine, in a stereoselective manner (Uzunov et al., 1996; Matsumoto et al., 1999; Serra et al., 2001; Pinna et al., 2004). These findings have a human correlate as CSF levels of this neurosteroid are restored in depressed patients following SSRI treatment in a manner which correlates with improved symptomatology (Romeo et al., 1998; Uzunova et al., 1998). Crucially, fluoxetine increased the levels of neurosteroids independently of any effect upon serotonin reuptake and on a rapid time scale (i.e. ∼30 min) that is not compatible with that associated with the clinical improvements reported for SSRI treatment (i.e. ∼3 weeks; Uzunov et al., 1996; Guidotti and Costa, 1998; Pinna et al., 2004). Such findings have thus raised the suggestion that an enhancement of GABA_A_R-mediated inhibition may contribute to the anxiolytic actions of these antidepressants (Longone et al., 2008). The mechanism by which fluoxetine and other SSRIs (e.g. paroxetine) increase the levels of 5α3α-THPROG was initially postulated to involve modulation of 3α-HSD activity (Uzunov et al., 1996; Griffin and Mellon, 1999). However, a direct interaction between SSRIs and 3α-HSD remains controversial as subsequent studies failed to support the initial findings (Trauger et al., 2002).

Establishing with confidence whether the increase in neurosteroid levels associated with SSRI treatment contributes to the therapeutic action of these antidepressants in humans has proved difficult to assess and remains unclear. Indeed, although various SSRIs (e.g. fluoxetine, paroxetine) elevate neurosteroid levels and alleviate depressive symptoms, a number of other pharmacological and non-pharmacological antidepressant treatments produce clinical improvements in patients suffering from major depressive disorders, independently of any effect upon the levels of 5α3α-THPROG (Padberg et al., 2002; Schule et al., 2003, 2006, 2014; Baghai et al., 2005). Challenging the clinical significance of increased neurosteroid levels ameliorating depression, 5α3α-THPROG levels increased in both responders and non-responders, following treatment with the antidepressant mirtazapine (Schule et al., 2006). Thus, it remains unclear whether the normalisation of 5α3α-THPROG levels following fluoxetine treatment is actively involved in alleviating depressive symptoms, or is simply a pharmacological by-product associated with clinical benefit. Nevertheless, the recent observation that the 18 kDa-translocator protein (TSPO) is the target of novel anxiolytic drugs (e.g. XBD173), that stimulate neurosteroidogenesis to produce anxiolytic effects, without the side-effect profile typically associated with classical BDZs (e.g. sedation, tolerance), suggests that neurosteroids and/or neurosteroidogenic compounds may still offer a promising approach for the treatment of affective disorders.

### 7.2 Hippocampal neurogenesis and stress: a role for GABA _A_R and neurosteroids and relevance to stress-associated psychopathology and treatment

Over recent years a relatively novel, but increasingly prevalent avenue of research aimed at understanding the pathophysiology of cognitive and major depressive disorders has focused upon the mechanisms regulating adult hippocampal neurogenesis. Adult neurogenesis is a complex multistep process that occurs in the mammalian hippocampus, striatum and olfactory bulb (Kempermann et al., 2004; Young et al., 2011), and appears, in animal models, to be particularly sensitive to physiological and environmental conditions, including acute or chronic stressor exposure (Cameron and Gould, 1994; Czeh et al., 2002; Heine et al., 2004; Oomen et al., 2007).

In this context, the role of GABA_A_R-mediated signalling in the proliferation, integration and survival of newborn hippocampal neurones may be of particular significance (Tozuka et al., 2005; Ge et al., 2006, 2007; Duveau et al., 2011). In common with neonates, newborn neurones in the adult hippocampus undergo a stereotypical integration process, where they initially receive tonic GABA_A_R activation followed by the generation of GABA_A_R-mediated synaptic inputs and finally glutamate-mediated synaptic inputs (Ge et al., 2007; Ben-Ari, 2002). Furthermore, electrophysiological analysis has revealed that GABA depolarises immature neural progenitor cells for the first two–three weeks of their neuronal development, an action that is crucial for normal structural and functional maturation as well as network integration (Tozuka et al., 2005; Ge et al., 2006). The GABAergic effects on cell proliferation, initial migration and early dendritic arborisation are mediated at least in the hippocampus by α4-containing GABA_A_Rs, whilst α2-GABA_A_Rs regulate the position of newborn neurones within the DG and the late maturation of their dendritic processes (Duveau et al., 2011). Stress in adult rodent models can induce suppression of neurogenesis, which is reversible following the cessation of stressor exposure or pharmacological treatment (Heine et al., 2004; Oomen et al., 2007). However, early-life stress-induced inhibition of neurogenesis appears to persist throughout life (Korosi et al., 2012). Thus, various models of prenatal stress exhibit cognitive and emotional deficits that associate with a long lasting impairment of hippocampal neurogenesis (Lemaire et al., 2000; Lucassen et al., 2009; Korosi et al., 2012). Although postnatal stress produces more variable results, in general similar deficits in hippocampal neurogenesis appear to prevail. Importantly, the effect of early life environment upon adult neurogenesis and the responsivity to novel experiences in adulthood appear to be dependent upon a number of factors including the age at which neurogenesis is determined, the frequency of the postnatal stressor (i.e. being either single or recurrent) and the gender (reviewed in Korosi et al., 2012; Loi et al., 2014). For example, exposure to a recurrent maternal separation paradigm during postnatal development impaired stress-induced plasticity of hippocampal neurogenesis in adulthood (Mirescu et al., 2004), whilst exposure to a single prolonged postnatal (P3) stress resulted in a suppression of adult hippocampal neurogenesis, impaired spatial learning, yet an enhancement of contextual learning in a stressful environment (Oomen et al., 2010). Interestingly, evidence from animal and clinical studies lends considerable correlative support to the notion that new neurones are required in the adult brain for antidepressant efficacy, but their role in the pathogenesis of mood disorders remains to be fully elucidated (Eisch and Petrik, 2012; Petrik et al., 2012; Castren and Hen, 2013). Specifically, the chronic treatment of rodents with several classes of antidepressants has resulted in an increase in hippocampal neurogenesis on a time scale consistent with the clinical efficacy of antidepressant treatment in humans (Malberg et al., 2000). In non-human primates stress also decreased neurogenesis, an effect prevented by the concomitant administration of the antidepressant tianeptine (Czeh et al., 2001). In further support, ablation of hippocampal neurogenesis using genetic and radiological methods attenuated the neurogenic and behavioural effects of antidepressant treatment (Santarelli et al., 2003). However, it should be noted that the therapeutic actions of certain antidepressant compounds have been reported to occur in a neurogenesis-independent manner (Surget et al., 2008; Bessa et al., 2009). Moreover, there is a general consensus that ablation of hippocampal neurogenesis is not sufficient to induce a depressive or anxiety-like behaviour in rodents (reviewed in Petrik et al., 2012). Thus, despite evidence suggesting a role for new born hippocampal neurones in mediating the efficacy of certain antidepressant compounds, a clear and coherent model explaining the mechanisms whereby adult born neurones contribute to emotional behaviours and/ or to the pathogenesis of depressive disorders remains to be formulated (Eisch and Petrik, 2012; Kheirbek et al., 2012). Specifically, further studies are required to elucidate the relationship between the role of new-born hippocampal neurones in the regulation of emotional behaviours and to determine how stress-induced changes in neurogenesis are relevant to the pathogenesis of depressive disorders.

Nevertheless, because GABA-mediated signalling is an important component of adult neurogenesis through a process that appears largely to recapitulate the neuronal developmental processes of embryonic/ neonatal stages, the GABA-modulatory actions of neurosteroids may be relevant and of possible therapeutic interest. Consistent with this suggestion, 5α3α-THPROG does exhibit proliferative effects in rat hippocampal, and human cortical progenitor cells in culture (Wang et al., 2005), raising the prospect that the regenerative properties of these endogenous neuromodulators may be of therapeutic benefit (Wang et al., 2008). Support for this proposal is provided by the effects of 5α3α-THPROG upon hippocampal neurogenesis and cognitive function in a mouse model of Alzheimer’s disease (3xTgAD). Thus, treatment of 3xTgAD mice with 5α3α-THPROG dose-dependently normalised abnormal cell proliferation in the SGZ to levels found in wild type mice and reversed the hippocampal dependent cognitive deficits (Wang et al., 2010). Importantly, the 5α3α-THPROG-induced survival of neural progenitors in the 3xTgAD mice correlated strongly with improved memory performance in these mice, suggesting that early deficits in neurogenesis may indeed contribute to the cognitive decline associated with Alzheimer’s disease. A similar beneficial effect of neurosteroid treatment upon hippocampal neurogenesis can therefore be postulated in stress-related pathologies. In agreement, in a social isolation rodent model of chronic stress, 5α3α-THPROG treatment either concomitantly, or following the period of social isolation, prevented the development of depressive-like behaviours measured by the novelty supressed feeding (NSF) and the forced swim test (FST) and concomitantly normalised the stress-induced suppression of hippocampal neurogenesis (Evans et al., 2012). Importantly, the antidepressant effects of 5α3α-THPROG in the FST and NSF paradigms were additionally accompanied by a normalisation of stress-induced BDNF deficits in the hippocampus (Evans et al., 2012). Although a clear mechanistic link remains to be established, evidence for a positive correlative association between 5α3α-THPROG levels and BDNF mRNA expression have been reported in a number of stress-related brain regions including the hippocampus, PFC and amygdala (Pibiri et al., 2008; Nin et al., 2011). The increase in BDNF following neurosteroid treatment is intriguing in the contest of the recently reported treatment of resistant-type of depression with ketamine (Niciu et al., 2014). Specifically, ketamine has been suggested to produce antidepressant effects by increasing synaptogenesis through complex intracellular signalling cascades, which appear dependent upon the increased synthesis and release of BDNF (Autry et al., 2011; Duman and Aghajanian, 2012; Liu et al., 2012; Dwyer and Duman, 2013; Pilc et al., 2013; Zunszain et al., 2013). Thus, establishing the relationship between BDNF and level of neurosteroids such as 5α3α-THPROG, and the potential effects of neurosteroids upon synaptogenesis in limbic and forebrain regions warrants further studies.

## 8 Concluding remarks

Since the pioneering discovery by Purdy and colleagues that a mild acute stress induced the *de novo* synthesis of GABA_A_R-active neurosteroids in the rodent brain, converging evidence from the past two decades has deduced an important role for these modulators of neuronal excitability in the regulation and programming of the stress response. However, our understanding of the specific mechanisms whereby neurosteroids may act to regulate both the programming of the stress response and some of the adaptations that ensue after acute as well chronic stress challenges in adulthood requires much refinement.

Firstly, our knowledge of the molecular signals that mastermind the expression of the genes for the synthesis and degradation of neurosteroids both in health and disease is limited. Furthermore, the recent report documenting a female-selective association between SNPs in the aldo–keto reductase 1 (AKRC1) and anxiety levels (Quast et al., 2014) highlights the potential importance of gender, further emphasising the importance of focused investigations in this area.

Secondly, although GABA_A_Rs are established as selective molecular targets of the stress-induced pregnane neurosteroids such as 5α,3α-THPROG and 5α,3α-THDOC, in contrast to other GABA_A_R modulators e.g. benzodiazepines, a detailed understanding of the specific receptor subtypes mediating the constellation of neurosteroid behaviours is not known. However, the identification of the molecular determinants for neurosteroid binding at the GABA_A_R and the recent development of mice engineered to express neurosteroid-insensitive GABA_A_R isoforms should be informative in this regard (Durkin et al., 2011). A similar approach should uncover the role specific receptor isoforms play in mediating the action of neurosteroids, both during early programming of the stress response and in the regulation of neuronal and behavioural adaptations to acute and/or chronic stress challenges.

Lastly, epidemiological studies suggest stress-associated psychopathology to exhibit sex-bias and, for example, women are ∼2–3 times more likely to be affected by mood disorders during their reproductive years. Indirect evidence, as discussed above (Section 2.2 and 7: Payne et al., 2009; Brummelte and Galea, 2010), suggest a potential role of neurosteroids in the gender-selectivity of stress-related psychopathology. Future efforts in the preclinical arena should therefore be conducted in both female and male subjects. Although appreciating the caveats associated with work in animals, such studies are nevertheless likely to prove invaluable in identifying candidate mechanisms and genes on which future human clinical research should focus.
